# Arginine supplementation in prevention of necrotizing enterocolitis in the premature infant: an updated systematic review

**DOI:** 10.1186/1471-2431-14-226

**Published:** 2014-09-10

**Authors:** Kevin Mitchell, Alexander Lyttle, Harish Amin, Huma Shaireen, Helen Lee Robertson, Abhay K Lodha

**Affiliations:** Department of Paediatrics, University of Calgary, Alberta Children’s Hospital, Calgary, T2N2T9 AB Canada; Section of Neonatology, Department of Paediatrics, University of Calgary, Foothills Medical Centre, Calgary, AB Canada; Health Sciences Library, Health Sciences Centre, University of Calgary, Calgary, Canada; Department of Community Health Sciences, University of Calgary, Calgary, T2N2T9 AB Canada; Alberta Children’s Hospital Research Institute, University of Calgary, Calgary, Canada

**Keywords:** Necrotizing enterocolitis, L-arginine, Premature infant

## Abstract

**Background:**

Hypoxic-ischemic injury is thought to play a significant role in necrotizing enterocolitis (NEC). Nitric Oxide (NO) is the principal inhibitory neurotransmitter in the gut and is involved in regulation of mucosal blood flow and maintenance of mucosal integrity. NO is synthesized from L-arginine by NO synthases. Our primary objective was to determine the effectiveness of supplemental L-arginine versus placebo in prevention of NEC in preterm infants ≤ 34 weeks gestational age by systematic review of published randomized controlled trials (RCTs).

**Methods:**

This review included RCTs in which L-arginine was administered as a supplement to neonates to prevent NEC. Searches were conducted in OVID MEDLINE, EMBASE, PubMed, and CINAHL from their dates of inception to July, 2014. Inclusion criteria were informed parental consent, neonates born at ≤ 34 weeks gestation, and birth weight ≤ 1500 g. Exclusion criteria included neonates with severe congenital anomalies and inborn errors of metabolism. Incidence of NEC was the primary outcome measure. Whole data were analyzed by RevMan 5.1 (Update Software, Oxford, UK). Outcome data were analyzed to determine risk ratios, number needed to treat, confidence intervals, and test for overall effect.

**Results:**

Two trials including 425 neonates were eligible for this review. Of these, 235 neonates were included in the study. L-arginine had a 59% reduction in the incidence of stage II and III NEC (RR 0.41, 95% CI 0.20 to 0.85, NNT = 9) compared with placebo (P = 0.02). A similar finding was identified for all stages of NEC (60% reduction, RR 0.40, 95% CI 0.23 to 0.69, NNT = 5) (P = 0.001). At age 3 yrs, there was no significant difference between the 2 groups in terms of any neurodevelopmental disability (RR 0.65; 95% CI 0.23-1.83, P = 0.41).

**Conclusions:**

L-arginine supplementation appears to be protective in prevention of NEC in preterm infants and without any significant impact on neurodevelopmental outcomes at 36 months of corrected age. With the addition of the results of one more study to the literature, an intriguing role for L-arginine supplementation continues to gain support. However, large multi-centre RCTs are needed before this can become common practice.

**Electronic supplementary material:**

The online version of this article (doi:10.1186/1471-2431-14-226) contains supplementary material, which is available to authorized users.

## Background

Necrotizing enterocolitis (NEC) is the most common acquired gastrointestinal emergency in premature infants. It is characterized by ischemic necrosis of the intestinal mucosa, inflammation, invasion of enteric gas-forming organisms, and dissection of gas into the muscularis and portal venous system [1]. NEC occurs in 1–3 per 1000 live births and 1–7.7% of admissions to neonatal intensive care units (NICU) [2]. The mortality of NEC varies based on the birth weight of the affected infant and the NEC Stage (I, II, III) and ranges from 20-30%, with the greatest mortality among infants requiring surgical intervention [3, 4]. The pathogenesis of NEC remains elusive; however, it is likely the result of a multifactorial process in a susceptible host. Of particular interest is the role played by intestinal vascular resistance in the development of NEC [5–7]. Hypoxic-ischemic injury is thought to play a significant role [8]. Mesenteric blood flow in neonates may decline in the presence of extreme hypoxia and severe abdominal distension [9, 10]. The resulting increased mesenteric vascular resistance can lead to reduced intestinal oxygen extraction and subsequent mesenteric acidemia [9]. Mucosal injury is seen initially, which may result in mucosal necrosis with ulceration and tissue sloughing [8]. Reperfusion-induced tissue damage after a hypoxic-ischemic event can produce ongoing injury to the intestinal mucosa via cytotoxic vascular endothelial cell damage and cytotoxic effects on cells of oxygen free radicals [8, 11–15]. NEC is a complex and multifactorial disease. Various clinical studies revealed that inflammatory mediators especially TNFα, IL-1, platelet activating factor, and nitric oxide (NO), produced by enterocytes and macrophages may play a role in the pathogenesis of NEC [16].

Nitric oxide (NO) plays an important role in maintaining baseline vasodilator tone [17]. It is the principal inhibitory neurotransmitter in the gastrointestinal system inducing gut smooth muscle relaxation, and helps regulate mucosal blood flow, maintenance of mucosal integrity, and intestinal barrier function [18–20]. A number of animal model studies of bowel injury have demonstrated that inhibition of NO increases the area of intestinal damage [5, 20–24]. NO is synthesized from the amino acid L-arginine by NO synthases (NOS) [17, 25]. Continuous intravenous infusion with L-arginine markedly reduced intestinal injury in a neonatal pig model of NEC [26]. Plasma arginine concentrations are decreased in premature infants with NEC [27, 28].

A Cochrane review of the role of L-arginine based on one study showed a reduction of NEC in premature neonates [29]. However, due to the small number of neonates in that study and without further evidence from other RCTs, the role of prophylactic L-arginine did not become a common practice in modern NICUs [30]. There is one more study published since the previous review [31]. The primary objective of this systematic review was to use all available data, including those from recently published randomized trials, to evaluate the effectiveness of supplemental L-arginine versus placebo in the prevention of necrotizing enterocolitis in preterm infants.

## Methods

The search strategy of the Cochrane Neonatal Review Group was used. The systematic review reporting follows the Preferred Reporting Items for Systematic Reviews and Meta-Analyses (PRISMA) [32].

### Search strategy for identification of studies

Searches were conducted in OVID MEDLINE, EMBASE, PubMed, and CINAHL from their date of inception to July 14, 2014, restricted to English language and human studies. The search strategy was developed jointly by the lead investigator (AKL) and a medical librarian (HLR) for OVID MEDLINE using exploded MeSH terms and keywords for premature infants, necrotizing enterocolitis, and L-arginine. This strategy was translated for EMBASE, PubMed, and CINAHL (HLR). Trials in which L-arginine supplementation was used prophylactically to prevent NEC in preterm neonates were included. References from previous reviews were also examined. All studies published in the English language were included in the study.

Search strategy: Controlled vocabulary (MeSH terms), keywords, and text words used: Infant, premature; necrotizing enterocolitis; L-arginine; neonatal intensive care; neonatal intensive care units; neonate. We identified relevant studies also by citation tracking. Experts in the field were also contacted to improve the search strategy. (Additional file 1).

### Eligibility criteria

Randomized controlled trials that compared L-arginine to control or placebo to use as a prophylactic agent to prevent NEC were included. Criteria for subject inclusion included neonates born at ≤ 34 weeks’ gestation, and with birth weight ≤ 1500 g. Exclusion criteria included neonates with severe congenital anomalies and inborn errors of metabolism. The selection of relevant studies was by consensus.

### Study identification and data extraction

All abstracts and published studies were independently identified and assessed for inclusion by two reviewers (KM, AL). Full papers were retrieved and checked for inclusion criteria. Each reviewer separately extracted data using the standardized Neonatal Cochrane group data abstraction forms. A third reviewer (HS) entered data into RevMan 5.1 (Update Software, Oxford, UK) and another reviewer cross-checked the printout against his/her data abstraction forms. The information was compared and all differences were resolved by consensus.

### Methodological quality

The methodological quality of the studies was assessed by two reviewers using the risk of bias assessment tool as endorsed by the Cochrane Neonatal Review Group and van Tulder’s guidelines [33]. The Cochrane Neonatal Review Group assessment included sequence generation, allocation concealment, blinding of outcome assessment, completeness of assessment, selective reporting bias and likelihood of other biases. van Tulder’s instrument is designed to assess internal validity of clinical trials and should include 11 items. Trials fulfilling six or more items were considered to be of high quality.

### Outcome measures

The incidence of all stages of NEC was the primary outcome measure. Secondary outcomes measured were stages II and III NEC, mortality in patients with NEC, incidence of respiratory distress syndrome (RDS), incidence of intraventricular hemorrhage (IVH), and neurodevelopmental outcomes at 36 months of corrected age. Neurodevelopmental disabilities were considered present if a child had any of cerebral palsy, mental retardation, blindness or deafness.

**Cerebral palsy:** (CP) refers to a non-progressive disability of movement and posture and was diagnosed on the basis of abnormal muscle tone and reflexes on the physical and neurological examination.

**Cognitive Delay:** Delayed cognitive function was diagnosed if there was a cognitive score >2 SD below the mean on age-appropriate standardized testing.

**Blindness:** Considered present if the infants had bilateral blindness with corrected visual acuity of <20/200 in the better eye.

**Deafness:** Defined as a bilateral sensorineural loss requiring amplification.

### Statistical analysis

The whole data were analyzed with Review Manager software (RevMan 5.1; Cochrane Centre) using Mantel-Haenszel method and fixed-effect model. Statistical analysis included relative risk ratios (RRs), number needed to treat (NNT) for dichotomous outcomes and weighted mean difference (WMD) for continuous outcomes. All estimates of treatment effects were reported with 95% confidence intervals (CI). Heterogeneity was assessed using a χ^2^-test and P-values lower than 0.05 were interpreted as being statistically significant.

## Results

**Figure 1:**
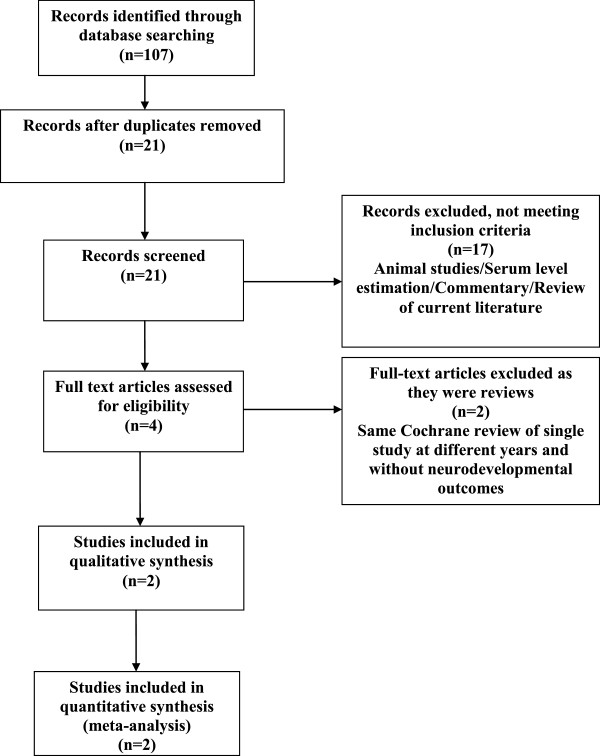
**Study selection for systematic review.**

Twenty-one studies were identified as being potentially relevant to this systematic review (Figure 1). Seventeen studies were excluded as they did not meet all the inclusion criteria. Two of the remaining four studies were systematic reviews by the same author but published in different versions of the Cochrane library based on one randomized, controlled trial without any revision. The remaining two studies were analyzed and data were compiled (Table 1).

**Table 1:** **Characteristics of included studies**

Study ID	Methods	Participants	Interventions	Outcomes	Allocation concealment
Amin et al.[30]	Randomized, double-blind, placebo-controlled, intention to treat basis	Total 254 infants eligible for the study	Study group: 1.5 mmol/kg per day L-arginine added to TPN. Once enteral feeds >40% TFI, L-arginine supplemented enterally	Primary outcome – NEC, all stages	Adequate
		Total 152 enrolled			
	Masking of allocation – Yes	Total 150 infants followed up, 1 died before commencing the study supplement, 1 was removed for IVH Grade ≥2	Control group: normal saline (same volume)		
	Masking of intervention – Yes				
	Masking of outcome assessment – Yes	Excluded patients with severe congenital anomalies, congenital non-bacterial infection, evidence of IVH Grade ≥2 on cranial ultrasound by day 3 of life, conjugated hyperbilirubinemia, evidence of an inborn error of metabolism, exchange transfusion during the study period, or with pre-existing kidney failure			
	Completeness of follow-up – Yes	Inclusion criteria – birth weight ≤1250 g and gestational age ≤32 weeks			
Polycarpou et al. [31]	Randomized, double-blind, placebo controlled	Total 171 infants eligible for the study	Study group: 1.5 mmol/kg per day liquid BID with NG feeds, from day 3–28 after birth.	Primary outcome – NEC	Adequate
	Masking of allocation – Yes	Total 83 enrolled	Control group: 5% glucose in equivalent volume		
	Masking of intervention – Yes	Total 83 infants followed up			
	Masking of outcome assessment – Yes	Excluded patients with severe congenital anomalies or inborn errors of metabolism.			
	Completeness of follow-up – Yes	Did not exclude patients with IVH Grade Stage ≥ 2			
		Inclusion criteria – birth weight ≤ 1500 g and gestational age ≤ 34 weeks			

*Abbreviations*: *IVH* intraventricular hemorrhage, *NEC* necrotizing enterocolitis, *NG* Nasogastric, *TFI* total fluid intake, *TPN* total parenteral nutrition.

### Methodological quality of included studies

One study scored 11 on the van Tulder qualitative assessment instrument and the other study scored 10, therefore, both were high quality studies (Table 1) [30, 31].

**Figure 2:**
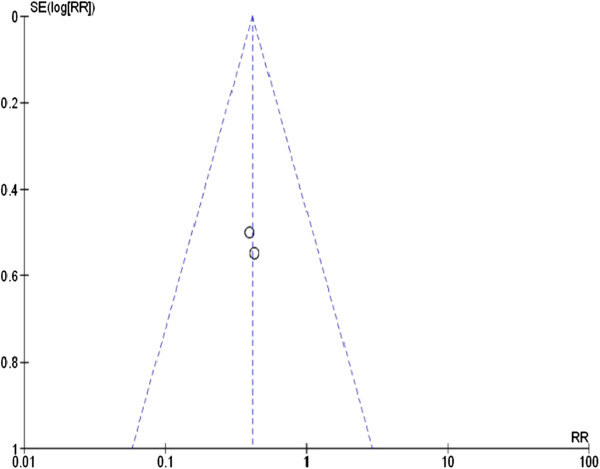
**Funnel plot to assess publication bias.** Each circle represents one study. Publication bias was not detected.

Two studies were included in the final analysis (Table 1). The efficacy of prophylactic L-arginine supplementation to prevent necrotizing enterocolitis in neonates was studied in both trials. One study administered L-arginine intravenously until enteral feeds reached a predetermined level of the total daily fluid intake, after which point L-arginine was supplemented enterally, while the other focused solely on enteral L-arginine administration [30, 31]. The number of patients varied between the studies; however, the follow-up period was the same. The patients’ characteristics were similar in both treatment and control groups (Table 2). The funnel plot is shown in Figure 2. This plot did not show any publication bias.

**Table 2:** Demographic data of enrolled neonates*

	L-arginine group		Placebo group	
	Amin et al. N = 75	Polycarpou et al. N = 40	Amin et al. N = 77	Polycarpou et al. N = 43
Male sex, n (%)	46 (61)	17 (42.5)	43 (56)	19 (44.2)
Birth weight, g, mean	952	1168	955	1127
Gestational age, wk, mean	27.4	29.2	27.6	28.8
Caesarian section, n (%)	ND	30 (75)	ND	32 (74.4)
Vaginal delivery, n (%)	ND	10 (25)	ND	11 (25.6)
IUGR, n (%)	5 (7)	16 (40)	7 (9)	14 (32.6)
Maternal antibiotics during labor, n (%)	45 (60)	14 (35)	50 (65)	18 (42)
Breast milk, n (%)	ND	7 (17.5)	ND	5 (11.6)
Preterm formula, n (%)	ND	33 (82.5)	ND	38 (88.4)
Apgar score at 5 minutes, median	8	8	7	8
Antenatal steroids, n (%)	66 (88)	32 (80)	66 (86)	34 (79.1)
IVH at study entry grade <2	9 (12)	ND	12 (16)	ND
Postnatal steroids, n (%)	23 (31)	ND	15 (19)	ND
Hypotension after birth, n (%)	30(40)	ND	24 (31)	ND
Umbilical arterial cord pH, median	7.25	ND	7.27	ND
Umbilical arterial cord Base excess	-4.3	ND	-3.8	ND
Umbilical artery catheter, n (%)	50 (67)	ND	57 (74)	ND

Values are presented as No. (%) unless otherwise indicated, *P-values = Non-significant.

*Abbreviations*: *IUGR* Intrauterine growth restriction, *IVH* Intraventricular hemorrhage, *ND* No data.

**Figure 3:**
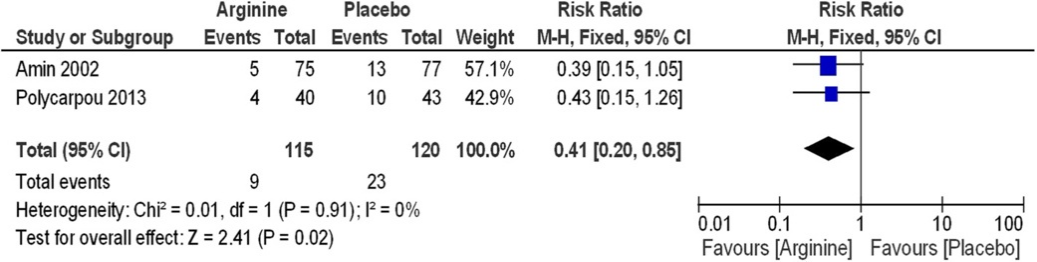
**L-arginine supplementation prevents stage II and III necrotizing enterocolitis in premature infants.**

**Figure 4:**
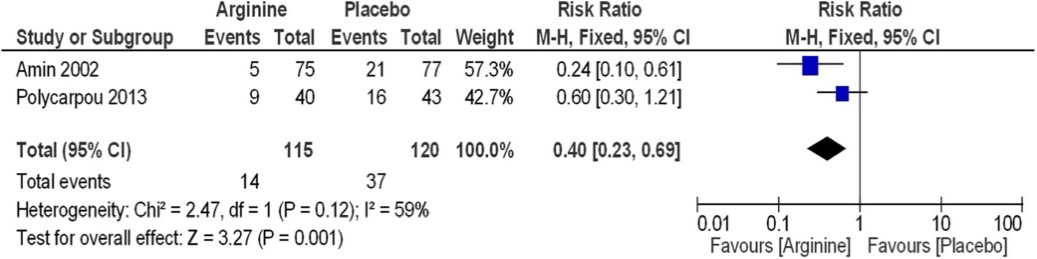
**L-arginine supplementation prevents necrotizing enterocolitis (all stages) in premature infants.**

The meta-analysis of the trials revealed that neonates who had received prophylactic supplemental L-arginine had a 59% reduction in the incidence of stage II and III NEC (RR 0.41, 95% CI 0.20 to 0.85; I^2^ = 0%) compared with placebo (P = 0.02) (Figure 3) and NNT was 9. Statistical significance was also present when comparing the L-arginine-supplemented group and the placebo group with respect to incidence of all stages of NEC (Figure 4) and NNT was 5. A 60% reduction in the incidence of NEC was noted in the L-arginine supplemented group (RR 0.40, 95% CI 0.23 to 0.69; I^2^ = 59%) (P = 0.001).

**Figure 5:**
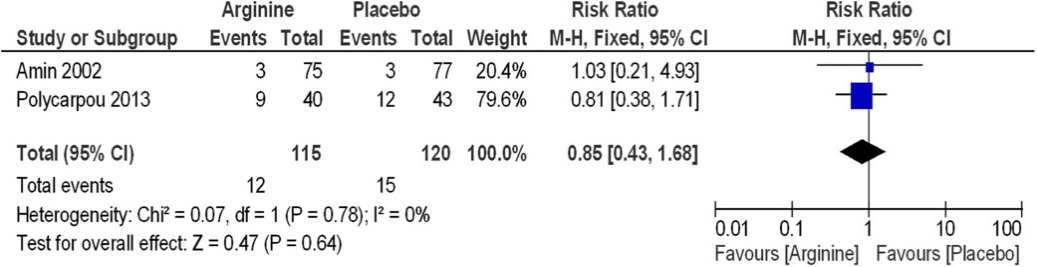
**Supplementation with L-arginine to prevent necrotizing enterocolitis in premature infants has no statistically significant difference on intraventricular hemorrhage incidence between study groups.**

**Figure 6:**
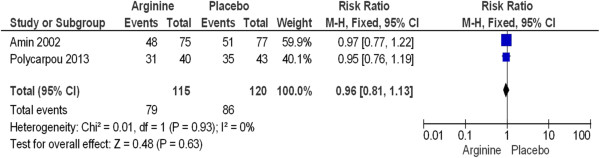
**Supplementation with L-arginine to prevent necrotizing enterocolitis in premature infants has no statistically significant difference on respiratory distress syndrome incidence between study groups.**

**Figure 7:**
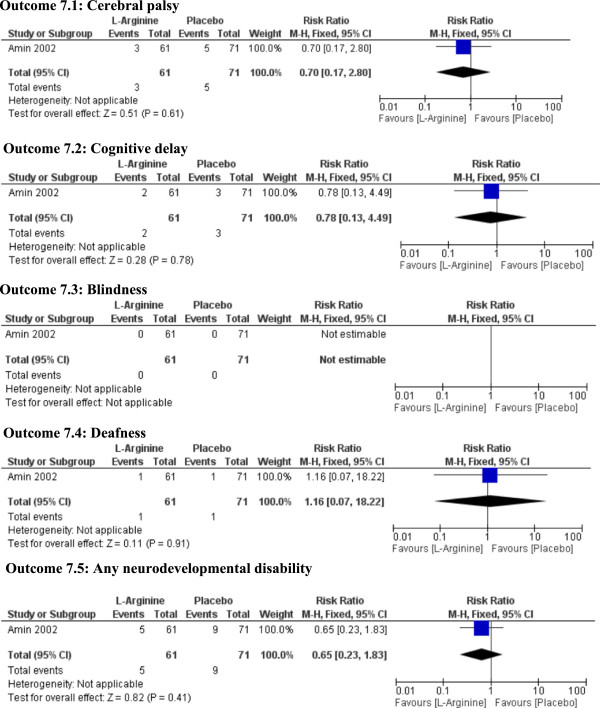
**Neurodevelopmental outcomes at 36 months corrected age.**

The incidence of intraventricular hemorrhage grades III and IV (Figure 5) (RR 0.85, 95% CI 0.43 to 1.68, P = 0.64) and respiratory distress syndrome (Figure 6) (RR 0.96, 95% CI 0.81 to 1.13, P = 0.63) were not statistically significant between groups (Table 3). Mortality due to NEC was also not statistically significant. Neurodevelopmental outcomes are shown in Figure 7.

**Table 3:** **Secondary outcomes**

Name of study	Outcome	Arginine group	Placebo/control	RR, 95% CI
Amin et al. [30]	RDS	48/75 (64)	51/77 (66)	0.97 (0.77-1.22)
Polycarpou et al. [31]	RDS	31/40 (77.5)	35/43 (81.4)	0.95 (0.76-1.19)
Amin et al. [30]	IVH grade III and IV	3/75 (4)	3/77 (4)	1.03 (0.21-4.93)
Polycarpou et al. [31]	IVH grade III and IV	9/40 (22.5)	12 (27.9)	0.81 (0.38-1.71)
Amin et al. [30]	Total PDA	46/75 (61)	45/77 (58)	1.13 (0.59-2.16)
Amin et al. [30]	PDA treated with indomethacin	33/75 (44)	38/77 (49)	0.89 (0.63-1.25)
Amin et al. [30]	PDA treated surgically	15/75 (20)	13/77 (17)	1.18 (0.61-2.32)
Amin et al. [30]	Sepsis	9/75 (12)	11/77 (14)	0.84 (0.37-1.91)
Amin et al. [30]	Hypotension after 24 h age	8/75 (11)	8/77 (10)	1.03 (0.37-2.90)

Values are presented as No. (%) unless otherwise indicated.

*Abbreviations*: *IVH* Intraventricular hemorrhage, *ND* No data, *PDA* Patent ductus arteriosus, *RDS* Respiratory distress syndrome.

## Discussion

The analysis of this updated systematic review of the RCTs conducted in premature infants receiving L-arginine as a prophylactic agent for prevention of NEC showed a statistically significant reduction in the incidence of stage II and III NEC (P = 0.02) (NNT = 9) and all stages of NEC (P = 0.001) (NNT = 5) in preterm infants supplemented with L-arginine compared to those in the placebo group. Given the NNT for both stage II and III NEC and all stages of NEC, this certainly makes the prospect of L-arginine having a role in the routine care of premature neonates an interesting one. In addition, with the absence of significant side effects and a reduction in both medical and surgical NEC, L-arginine may have a prominent role in upcoming years.

Our results demonstrate a statistically significant reduction in the incidence of stage II and III NEC. This was initially suggested by Amin et al.; however, did not reach statistical significance (P = 0.077) [30]. The results from Polycarpou et al. were also non-significant [31]. The significant P-value that was found in our study is likely in large part due to the increased proportion of cases of stage III NEC in Polycarpou et al. compared to Amin et al. and the larger sample size achieved from pooling the studies. As such, our results support Amin et al.’s previous findings that were suggestive of a reduction in the incidence of NEC [30]. Polycarpou et al.’s study did not show statistical significance for all stages of NEC, but did note a statistically significant reduction in the incidence of stage III NEC [31].

There were no statistically significant differences between both groups with respect to secondary outcomes (Table 3). Regarding IVH, Polycarpou et al. did not exclude neonates with IVH grades III and IV, as they were in Amin et al.’s study [31, 30]. As such, a prominent difference was noted in the proportion of infants with grades III and IV IVH when data were compared: 4% of Amin et al.’s total sample vs. 25% of Polycarpou et al.’s total sample [30, 31]. When the data were combined and compared against placebo, a statistically significant difference was not found (P = 0.64).

We demonstrate in our systematic review that L-arginine supplemented infants in one RCT for prevention of NEC did not have any difference in the long term neurodevelopmental outcomes at the age of 36 months of corrected age compared with those who received placebo [34].

NO plays a key role in intestinal epithelial injury in NEC. Ford and his co-investigators have established the role of iNOS-derived NO in NEC and also found an upregulation of iNOS mRNA and protein in infants undergoing laparotomy for NEC, as compared to infants those were undergoing for resection of intestine due to other reasons [6]. NO is an endothelial-derived relaxing factor – a potent, short-lived vasodilator. NO also modulates various physiological processes including tissue homeostasis, neurotransmission, and inflammation. Nitric oxide is a product of NO synthase (NOS) which converts arginine and oxygen into NO and citrulline. There are three isomers of NOS and each coded by different genes. Endothelial NOS (eNOS) and neuronal NOS (nNOS) isoforms are expressed at low levels and these enzymes produce a small amount of NO. Both of these isoforms are activated by calmodulin. The third isoform is calcium-independent and is known as inducible NOS (iNOS) and binds to calmodulin with a very good affinity. iNOS isoforms are produced at high levels during periods of inflammation. During expression of iNOS, there is further production of NO in nanomolar to micromolar concentrations. The reaction of NO with superoxide leads to the production of peroxynitrite, a potent oxidant. These molecules further lead to cytopathic effects and result in enterocyte apoptosis or necrosis, impairment of enterocyte proliferation, and epithelium restitution through enterocyte migration. Tissue injury and repair initiates the inflammatory cascade, leading to the classical picture of NEC [6, 35, 36].

The limited *de novo* arginine production capacity in neonates makes arginine an essential amino acid in early life. In these two studies, L-arginine in premature infants was supplemented with the intention of increasing NO synthesis with the rationale that NO’s role as a vasodilator would be protective to the gut through prevention of ischemic injury [30, 31]. Interestingly, while only a fraction of arginine metabolism enters the NOS pathway to produce NO, it appears as though this small proportion of the overall body arginine lends substantially to the prevention of intestinal ischemia, likely via regulating mesenteric blood flow.

The strengths of this updated systematic review are the inclusion of a recent trial, increased power based on sample size, and detailed subgroup analyses. The current analysis provides evidence in the favor of prophylactic use of L-arginine in premature infants to prevent NEC.

This review included only two small RCTs with a small number of subjects. The limitation of the two included studies was overcome by conducting this systematic review. Additionally, one of the two studies was underpowered. Despite this, statistically significant reductions in the incidence of stage II and III NEC, as well as all stages of NEC, were noted, with p-values of less than 0.05.

## Conclusions

Our study revealed that L-arginine has a significant role in reducing the incidence of medical and surgical NEC in modern NICUs without impact on long-term neurodevelopmental outcomes at 36 months of corrected age. However, in the absence of large multi-centre, randomized, controlled trials, the use of supplemental L-arginine in an effort to prevent necrotizing enterocolitis in preterm neonates has not become routine practice.

### Implications for practice

Given the significant morbidity and mortality associated with medical and surgical NEC, a preventative measure to reduce the incidence and severity of the disease would be a welcomed addition to routine NICU care. Considering the findings of this study, particularly the NNT of 9 for stages II and III NEC and the NNT of 5 for all stages of NEC, an intriguing role for L-arginine supplementation continues to gain support.

### Future research

With the addition of the results of this study to the literature, L-arginine supplementation continues to gain support and will become the basis for a future large clinical trial. We believe that large multi-centre RCTs are needed before such supplementation can become common practice.

## Authors’ information

KM: MD, Neonatal-Perinatal Medicine Fellow, University of British Columbia, Children’s and Women’s Health Centre of British Columbia, Vancouver, British Columbia, Canada

AL: MD, Paediatric Allergy & Immunology Fellow, University of British Columbia, Children’s and Women’s Health Centre of British Columbia, Vancouver, British Columbia, Canada

HA: MBBS, FRCPC, Staff Neonatologist, Director, NICU, South Health Campus, Alberta Health Services; Associate Professor, Department of Pediatrics, University of Calgary, Calgary, Alberta, Canada

HS: MD, FCPS (Ped). Third-year Neonatal-Perinatal Medicine Fellow, University of Calgary, Foothills Medical Centre, Calgary, Alberta, Canada

HLR: MLIS, BA, Liaison Librarian, Clinical Medicine, Health Sciences Library, Health Sciences Centre, University of Calgary, 3330 Hospital Drive NW, Calgary, Alberta, Canada

AKL: MBBS, MD, DM, MSC, Staff Neonatologist, Foothills Medical Centre, Alberta Health Services, Clinical Epidemiologist; Assistant Professor, Department of Pediatrics and Department of Community Health Services, Alberta Children’s Hospital Research Institute, University of Calgary, Calgary, Alberta, Canada

## Electronic supplementary material

Additional file 1: **Database: Ovid MEDLINE(R) In-Process & Other Non-Indexed Citations and Ovid MEDLINE(R) <1946 to Present>.** (DOC 24 KB)

## Abbreviations

CI: Confidence interval
CINAHL: Cumulative Index to Nursing and Allied Health Literature
CP: Cerebral palsy
IUGR: Intrauterine growth restriction
IVH: Intraventricular hemorrhage
NEC: Necrotizing enterocolitis
NICU: Neonatal intensive care unit
NO: Nitric oxide
NOS: Nitric oxide synthases
RCT: Randomized, controlled trial
RDS: Respiratory distress syndrome
VLBW: Very low birth weight
WMD: Weighted mean difference.
