# Supplementary material for: Arginine supplementation in prevention of necrotizing enterocolitis in the premature infant: an updated systematic review
Source: BMC Pediatr. 2014 Sep 10;14:226. doi: 10.1186/1471-2431-14-226 (PMC4166475; doi:10.1186/1471-2431-14-226)
Supplement: Supplementary file 1 — Additional file 1: Database: Ovid MEDLINE(R) In-Process & Other Non-Indexed Citations and Ovid MEDLINE(R) <1946 to Present>. (DOC 24 KB) [file 12887_2014_1149_MOESM1_ESM.doc]

Additional file 1

Search for: 21 not 24

Results: 17

Database: Ovid MEDLINE(R) In-Process & Other Non-Indexed Citations and Ovid MEDLINE(R) <1946 to Present>
Search Strategy:
--------------------------------------------------------------------------------
1     exp Infant, Premature/ (41465)
2     exp Infant, Newborn/ (497323)
3     premature infant$.tw. (16042)
4     prematurity.tw. (14236)
5     newborn$.tw. (127341)
6     neonatal.tw. (150946)
7     neonate$.tw. (65229)
8     exp Intensive Care, Neonatal/ (4329)
9     exp Intensive Care Units, Neonatal/ (9750)
10     neonatal intensive care.tw. (11841)
11     newborn intensive care.tw. (481)
12     or/1-11 (634284)
13     exp Arginine/ (50580)
14     l-arginine.mp. (30370)
15     l-arginine.rn,nm. (493)
16     13 or 14 or 15 (62022)
17     exp Enterocolitis, Necrotizing/ (1967)
18     necrotizing enterocolitis.tw. (4411)
19     necrotising [enterocolitis.tw](http://enterocolitis.tw/). (659)
20     17 or 18 or 19 (5391)
21     12 and 16 and 20 (27)
22     limit 21 to animals (13)
23     limit 21 to (animals and humans) (3)
24     22 not 23 (10)
25     21 not 24 (17)

***************************
